# Inhibition of Phosphoinositide 3-Kinase p110delta Does Not Affect T Cell Driven Development of Type 1 Diabetes Despite Significant Effects on Cytokine Production

**DOI:** 10.1371/journal.pone.0146516

**Published:** 2016-01-19

**Authors:** Ariana Barbera Betancourt, Juliet L. Emery, Asha Recino, F. Susan Wong, Anne Cooke, Klaus Okkenhaug, Maja Wallberg

**Affiliations:** 1 Department of Pathology, University of Cambridge, Tennis Court Road, CB2 1QP Cambridge, United Kingdom; 2 Laboratory of Lymphocyte Signalling and Development, Babraham Institute, Cambridge CB22 3AT, United Kingdom; 3 Diabetes Research Group, Institute of Molecular and Experimental Medicine, Cardiff School of Medicine, Cardiff University, Cardiff CF14 4XN, United Kingdom; University of Rochester, UNITED STATES

## Abstract

Type 1 diabetes is caused by the destruction of insulin producing beta cells by the immune system. The p110δ isoform of PI3K is expressed primarily in cells of haematopoietic origin and the catalytic activity of p110δ is important for the activation of these cells. Targeting of this pathway offers an opportunity to reduce immune cell activity without unwanted side effects. We have explored the effects of a specific p110δ isoform inhibitor, IC87114, on diabetogenic T cells both *in vitro* and *in vivo*, and find that although pharmacological inhibition of p110δ has a considerable impact on the production of pro-inflammatory cytokines, it does not delay the onset of diabetes after adoptive transfer of diabetogenic cells. Further, we demonstrate that combination treatment with CTLA4-Ig does not improve the efficacy of treatment, but instead attenuates the protective effects seen with CTLA4-Ig treatment alone. Our results suggest that decreased IL-10 production by Foxp3^+^ CD4^+^ T cells in the presence of IC87114 negates individual anti-inflammatory effects of IC8114 and CTLA4-Ig.

## Introduction

Type 1 diabetes is an autoimmune inflammatory disease that is caused by immune cell mediated destruction of the insulin-producing beta cells in the pancreas. T cells are assumed to play a considerable role in the pathogenesis of this disease which comes from the demonstration that its strongest genetic risk is conferred by the HLA locus and other loci affecting the biology of these cells [1, 2]. Islet specific CD4^+^ and CD8^+^ cells mediate diabetogenesis in NOD mice [3] and have been identified in human type 1 diabetes patients [4–6]. Other cell types including B cells, dendritic cells and macrophages are also necessary for the initiation of the anti-islet immune responses (reviewed in [7]). Once the beta cell pool is destroyed by the immune system, insufficient amounts of insulin are produced to sustain glucose uptake into insulin dependent cells, notably muscle cells, causing wasting due to protein breakdown, even though high levels of glucose are present in the blood. The disease was fatal until Banting and Best discovered insulin in 1922 [8]. Although much has been learned about type 1 diabetes since then, no protocols for sustained cure or prevention have been discovered. Strategies attempting to abrogate immune responses have been successful in mouse models [7], and some have also resulted in delay of beta cell destruction in patients, such as the anti-CD3 treatment clinical trials [9, 10] and CTLA-4 Ig [11].

CTLA-4 Ig prevents the activation of T cells by inhibiting interaction with the co-stimulatory molecule CD28. However, CD28 blockade alone is unlikely to be sufficient to prevent autoimmunity due to the fact that memory and CD8^+^ T cells are not as dependent as naïve CD4^+^ T cells on CD28 costimulation. As none of the clinical trials have resulted in long term reversal of diabetes, it has been suggested that a combination of treatments targeting various stages of immune activation may be more successful [12]

A widely explored approach is the inhibition of key signalling enzymes involved in the activation and metabolism of immune cells. PI3Ks constitute a family of enzymes involved in cellular functions such as cell growth, proliferation, differentiation, motility, survival and intracellular trafficking [13]. The class I PI3Ks catalyse the final step to PIP3 by phosphorylating PI(4,5)P2 to become PI(3,4,5)P3. PIP3 activates Akt, which in turn leads to activation of mTOR and inhibition of Foxo. Of the four class I PI3K subunits, PI3K p110δ is uniquely expressed in immune cells [14]. Specific inhibition of this subunit therefore primarily affects cells of the immune system, leaving other cell types unchanged. p110δ inhibitors such as IC87114 have been used to inhibit p110δ activity *in vitro* and *in vivo*. It has been shown that IC87114 inhibits TCR-induced cytokine production by both naïve and effector T cells [15]. In addition to its effects on T cells, p110δ inhibitors also target other immune cells such as B cells, NK and myeloid cells (reviewed in [14, 16] The typical outcome of PI3Kδ inactivation is a reduction but not a complete abrogation of leukocyte functions, probably due to the overlapping functions with other PI3K members that are also present in leukocytes. IC87114 reduced disease severity in preclinical rodent models of rheumatoid arthritis, asthma, and allergy [17–19]. The PI3Kδ inhibitor idelalisib was approved in 2014 for the treatment of chronic lymphocytic leukaemia.

CD28 and p110δ act synergistically to provide full T-cell stimulation [20], and we hypothesised that dual inhibition of CD28 and p110δ may achieve more potent alleviation of pathologic immune responses than can be achieved with either inhibitor alone. To test this hypothesis, we used IC and CTLA-4 Ig, either alone or in combination, to inhibit signalling via p110δ, CD28 or both in naïve and activated T cells from non obese diabetic (NOD) mice and TCR transgenic mice on a NOD background, and monitored proliferation and cytokine production. In addition, we have investigated whether such a combination could limit the development of autoimmune diabetes in the NOD mouse model after the transfer of either naïve or activated islet reactive BDC2.5 CD4^+^ T cells.

## Materials and Methods

### Mice

Female NOD, NOD-*scid*, BDC2.5 NOD mice [21] NOD-CD2-GFP mice [22], G9C8 TCR transgenic NOD mice [23, 24] and NOD-Foxp3-GFP [25] were bred in the Department of Pathology, University of Cambridge and maintained under specific pathogen—free conditions. The mice are housed in individually ventilated cages with free access to standard chow and water. The facility is kept on a 12 hour light, 12 hour dark cycle. The humane endpoints for these experiments specify that any mouse that loses more than 15% of its body weight (compared to healthy littermates), or in other ways looks unwell and likely to exceed the Home Office standard of moderate severity must be culled. However, no mice used in this study required early culling. At the end the experiments, mice were culled using a CO2-chamber followed by dislocation of the neck. In cases where we were harvesting islets for transplantation it was important to maintain an intact bile duct, and death was instead confirmed through palpation of the chest to assess the absence of a heart beat.

### Ethics statement

This study was carried out in strict accordance with U.K. Home Office project licence regulations (Project Licence number 80/2442) after approval by the Ethical Review Committee of the University of Cambridge.

### PI3K-δ inhibitor

The PI3K p110δ inhibitor IC87114 was synthesized by Jonathan Clark (Babraham Institute) as described (D030 from patent WO 01/81346) [15]. For *in vivo* administration, IC87114 was dissolved in methyl cellulose 400 cps (Sigma) using a sonicator (Heat Systems Ultrasonics), and administered through oral gavage twice daily in 100μl at a dose of 30mg/kg body weight. This dose was chosen based on previous reports of its efficacy in vivo [17]. In our hands, a 30 mg/kg by gavage achieves ~2 μM 90 min post-administration and the drug is cleared from the blood 4–7 hours post administration. IC87114 is selective for p110δ at plasma concentrations of 5 μM [17].

### CTLA4-Ig

CTLA4-Ig (Abatacept) was provided by Bristol Myers Squibb (BMS). CTLA4-Ig was administered by intraperitoneal (ip) injection starting on day 0 with 500 μg, then 250μg every other day [26]. For *in vitro* assays, CLTA4-Ig was added to cultures at 100 ng/ml.

### Th1 differentiation for *in vitro* studies and adoptive transfer

CD4^+^CD25^-^ T cells (for *in vitro* studies) or CD4^+^CD62L^hi^ CD25^-^B220^-^ T cells (for adoptive transfer) were isolated by cell sorter from 5-week-old BDC2.5 TCR transgenic NOD mice and differentiated into Th1 cells by culturing them with plate bound anti-CD3 (2μg/mL), soluble anti-CD28 (10μg/mL), IL-2 (100u/ml), IL-12 (10ng/ml) and IFN-γ (100u/ml) for 4 days at 37°C with 5% CO_2_. Afterwards, the production of IFN-γ was checked by specific ELISA (R&D).

### T cell activation for functional assays

Cells were isolated from spleen and lymph nodes and cultured in IMDM with 10% fetal calf serum, 1% penicillin-streptomycin, and β-mercaptoethanol. 2x10^5^ total lymphocytes were stimulated as appropriate (see below) for 3 days in the presence or absence of rising concentrations of IC (0.6, 1.25, 2.5, 5 and 10μg/mL) at 37°C with 5% CO2. NOD mouse cells were stimulated with plate bound anti-CD3 (2μg/mL) and soluble anti-CD28 (10μg/mL), whereas cells isolated from BDC2.5 or G9C8TCR transgenic NOD mice were stimulated with BDC2.5 mimotope or insulin peptide insB 15–23, respectively. In other experiments, 2.5x10^5^ Th1 cells were cultured with 1x10^4^ APCs and BDC2.5 mimotope (0.5μg/mL) with or without increasing concentrations of IC as previously described for 72 hours. Cells cultured in the presence of the proliferative stimulus but without IC87114 were positive controls, whereas non-stimulated cells were negative controls.

### Proliferation and cytokine analysis

In all experiments proliferation was assessed by CFSE staining (5μM). After gating on CD4^+^ and/or CD8^+^ T cells, the percentage of proliferating cells in each population was determined. For cytokine analysis, supernatants were taken at the end of the time cultures and IFN-γ production or IL-10 was assessed by specific ELISA (R&D Systems). Levels of other cytokines were detected using a cytometric bead array (eBioscience). For intracellular cytokine staining, cells were washed and stimulated with PMA (50 ng/mL) and ionomycin (2000 ng/mL) for 5 hours. BFA (5μg/mL) was added for the last 3 hours. Afterwards, the staining of cell surface markers was performed. Cells were washed, fixed, permeabilized (intracellular cytokine staining kit, eBioscience), and stained for detection of IFN-γ.

### *In vitro* assessment of regulatory T cells

CD4^+^ CD25^+^ GFP^+^ cells from spleens and lymph nodes of 5-week-old Foxp3/GFP^+^ BDC2.5 TCR transgenic NOD mice were isolated by cell sorter (MoFlo, BD). Tregs were cultured with anti-CD3 (5 μg/mL), anti-CD28 (20μg/mL) and IL-2 (1000 u/ml) with or without IC87114 (5 and 10μM) for 72 hours. Proliferation was assessed by dilution of CFSE staining (5μM, Invitrogen) after gating on CD4^+^CD25^+^ cells. Supernatants were assessed for IL-10 by specific ELISA (R&D). Positive control cells were stimulated with anti-CD3/28 antibodies and IL-2 whereas negative control cells were not stimulated at all.

### Islet transplantation

Diabetes was induced in recipient mice (normal (WT), CD28-/-, p110δ^D910A/D910A^ and CD28-/-; p110δ^D910A/D910A^ double knockouts (DKOs)) by streptozotocin injection. All strains were backcrossed on the C57BL/6 background. Islets were prepared from MHC-mismatched donors (Cba1-C57BL/6 F1). Pancreatic islets were isolated through inflation of the pancreas via the bile duct [27], and islet transplantation was performed according to standard protocols [28]. Confirmed diabetic recipient mice received between 300 and 500 islets, giving approximately 15 islets per gram of body weight. We anaesthetised the recipient mice with isoflurane inhalation anaesthesia, and gave them sc temgesic for post-surgery analgesia. Islets were injected beneath the kidney capsule of female recipients that have been confirmed to be diabetic after the streptozotocin injection (Blood glucose level > 20 mM at two consecutive readings). Blood glucose was assessed three times per week for the period of graft survival, and daily at the onset of graft rejection—for a maximum of three days. Some mice which retained their grafts for longer than 100 days had the engrafted kidney removed at the end of the experiment to ascertain that the graft was responsible for the restoration of euglycemia.

### Adoptive transfer models

CD4^+^ T cells were isolated by cell sorting from spleen and lymph nodes of BDC2.5 TCR transgenic mice. 1x10^6^ CD4^+^ T cells were transferred by the ip route to 5-week old NOD-*scid* mice. Mice received IC87114 treatment (30mg/Kg) by oral gavage twice per day and/or CTLA-4 Ig administrated by the ip route at a concentration of 500μg/mL (the first time) and then 250μg/mL every other day from day 0 to 10. Positive control mice did not receive any treatment. After day 7, mice were monitored for the development of diabetes by measuring blood glucose or urine glucose levels with Diastix strips (Bayer) every day. In other experiments, 5x10^5^ Th1 cells (see above) were transferred by the ip route to 5-week old NOD mice. Mice received the same treatment as described above, but from day 0 to 5. After day 3, mice were monitored for the development of diabetes as previously described.

### Statistical analysis

Differences between groups were tested using the student t-test, significant p-values are indicated with * (p≤0.05), ** (p≤0.001) or *** (p≤0.0001). Differences between animals regarding diabetes incidence or recurrence after islet transplant were tested using the Log rank survival test, with actual p-values displayed within the relevant figure or legend. All analysis was performed using GraphPad Prism software.

## Results

### IC87114 inhibition of the PI3K p110δ subunit does not diminish T cell proliferation but has profound effects on cytokine production

**Fig 1:**
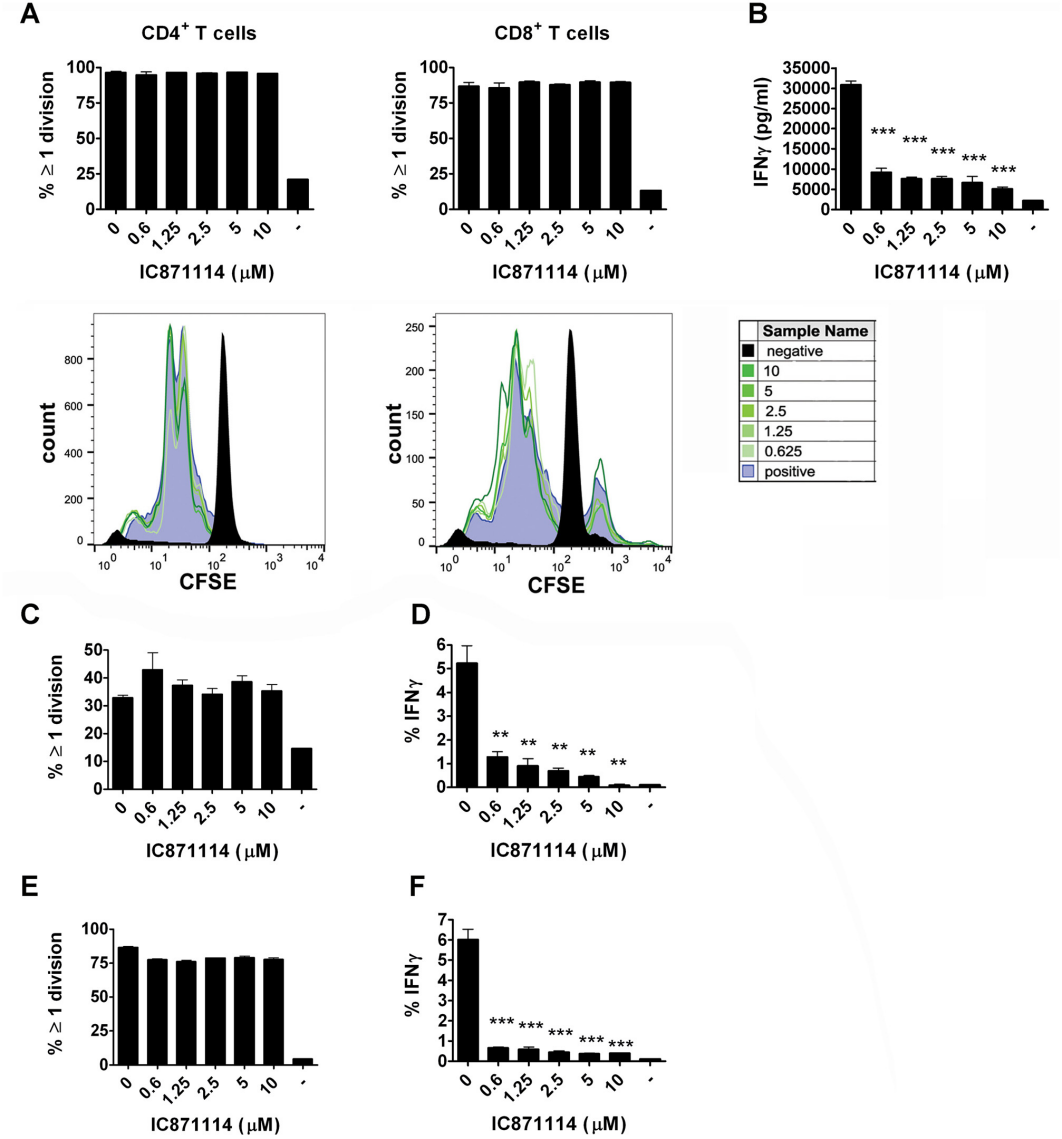
IC87114 blocks IFN-γ production by activated NOD cells. Cells isolated from the spleens and lymph nodes of regular NOD mice (A,B); BDC2.5 TCR transgenic NOD mice (C,D) or G9C8 TCR transgenic NOD mice (E,F) were stimulated with anti-CD3/28 antibodies (A, B), BDC2.5 mimotope (C, D) or insulin peptide (E, F), respectively, with or without increasing concentrations of IC87114 (0.6–10μM) for 72 hours. (A,C,E) Cells were stained with CFSE and after gating on CD4^+^ and/or CD8^+^ T cells, the percent of proliferating cells in each population was determined. (B) Supernatants were collected and IFN-γ production was assessed by specific ELISA (B) or intracellular staining (D, F). All data were expressed as the mean ± SD for triplicate samples, differences between groups were tested using the student t-test. The data is representative of at least three independent experiments.

We assessed the effects of increasing levels of IC87114 on T cells from diabetes-prone NOD mice in *in vitro* culture, looking at anti-CD3 and anti-CD28 induced proliferation of CD4^+^ T cells (Fig 1A, left hand panel) and CD8^+^ T cells (Fig 1A, right hand panel) and production of IFN-γ (Fig 1B). We found that the presence of IC87114 did not suppress proliferation of either CD4^+^ or CD8^+^ T cells (Fig 1A) but that the production of IFN-γ was severely impaired even at the lowest concentration of IC87114 tested (Fig 1A). Unaffected proliferation (as measured by % of cells divided ≥1 time) and suppressed IFN-γ production were seen both in islet specific TCR transgenic BDC2.5 CD4^+^ T cells [21] (Fig 1C and 1D), which recognise a posttranslationally modified peptide of chromogranin A [29], and in TCR transgenic G9C8 CD8^+^ T cells which recognise an insulin peptide [23, 24] (Fig 1E and 1F). We performed an extended analysis of the effects on cytokine production by IC87114, and found that increasing concentration of IC87114 suppressed all the pro-inflammatory cytokines assessed in supernatants from peptide-stimulated BDC2.5 CD4^+^ T cells, including IFN-γ, IL-2, GM-CSF, and IL-6, while there was a trend towards increased production of anti-inflammatory cytokine IL-10 and Th2 associated cytokines IL-4 and IL-5 (S1 Fig). Production of the cytokines that could be detected in supernatants from insulin peptide stimulated G9C8 CD8^+^ T cells was also decreased, with lower levels of IFN-γ, IL-2, TNF, IL-17, GM-CSF, IL-10 and IL-6 recorded even at the lowest concentrations of added IC87114 (S2 Fig). An interesting finding was that T cell proliferation was if anything increased in the presence of higher concentrations of IC87114, with more cells dividing several times (S3A Fig) and with BDC2.5 CD4^+^ T cells upregulating CD25 in response to increasing concentration of IC87114 (S3B Fig).

### Administration of IC87114 via oral gavage does not delay onset of diabetes after BDC2.5 cell transfer into NOD-*scid* recipients

**Fig 2:**
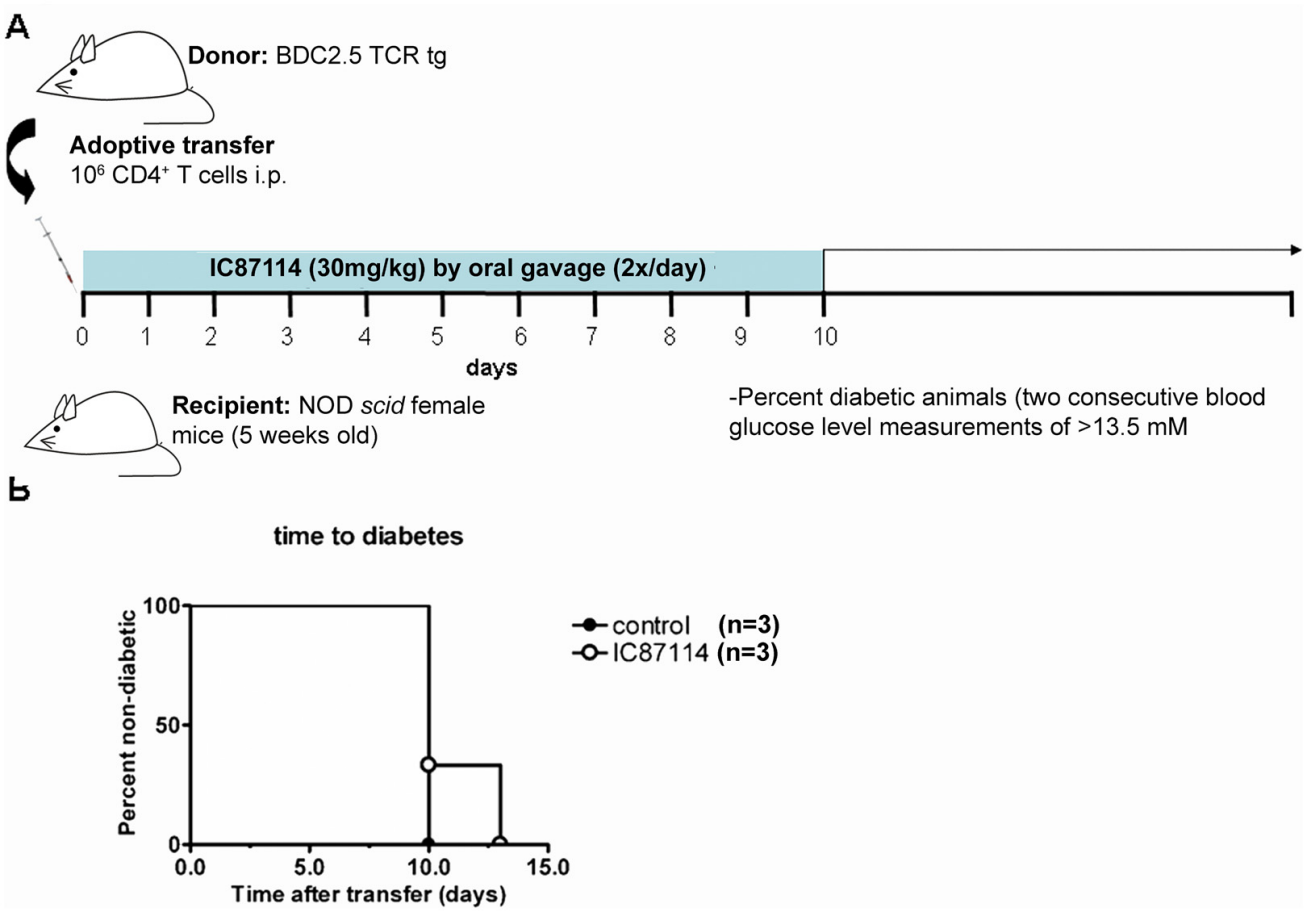
Administration of IC87114 does not prevent diabetes after adoptive transfer of naïve diabetogenic cells. CD4^+^ T cells were isolated by cell sorting from spleen and lymph nodes of BDC2.5 TCR transgenic mice. 1x10^6^ CD4^+^ T cells were transferred i.p. route to NOD-*scid* mice. Mice received IC87114 treatment (30mg/Kg) by oral gavage (2x/day) from day 0 to 10 (A). Positive controls are mice that received CD4^+^ T cells from BDC2.5 mice without any other treatment. After this time, blood glucose levels were checked every day, and mice were considered irrevocably diabetic and sacrificed when they reached two consecutive blood glucose levels >13mM (B).

As production of proinflammatory cytokines is central to the process whereby islet-specific T cells cause type 1 diabetes through incapacitating and killing beta cells [30], we wanted to test whether oral administration of IC87114 could prevent or delay onset of diabetes in NOD*scid* mice after transfer of potentially diabetogenic islet-specific BDC2.5 CD4^+^ T cells. We found that oral administration of 30mg/kg body weight twice per day (Fig 2A) had no effect of the development of diabetes after cell transfer, as all mice in both groups had developed diabetes by day 13 after transfer (Fig 2B).

### IC87114 suppresses IFN-γ production from Th1-differentiated islet specific effector T cells but does not stop them from causing diabetes after transfer into wt NOD recipients

**Fig 3:**
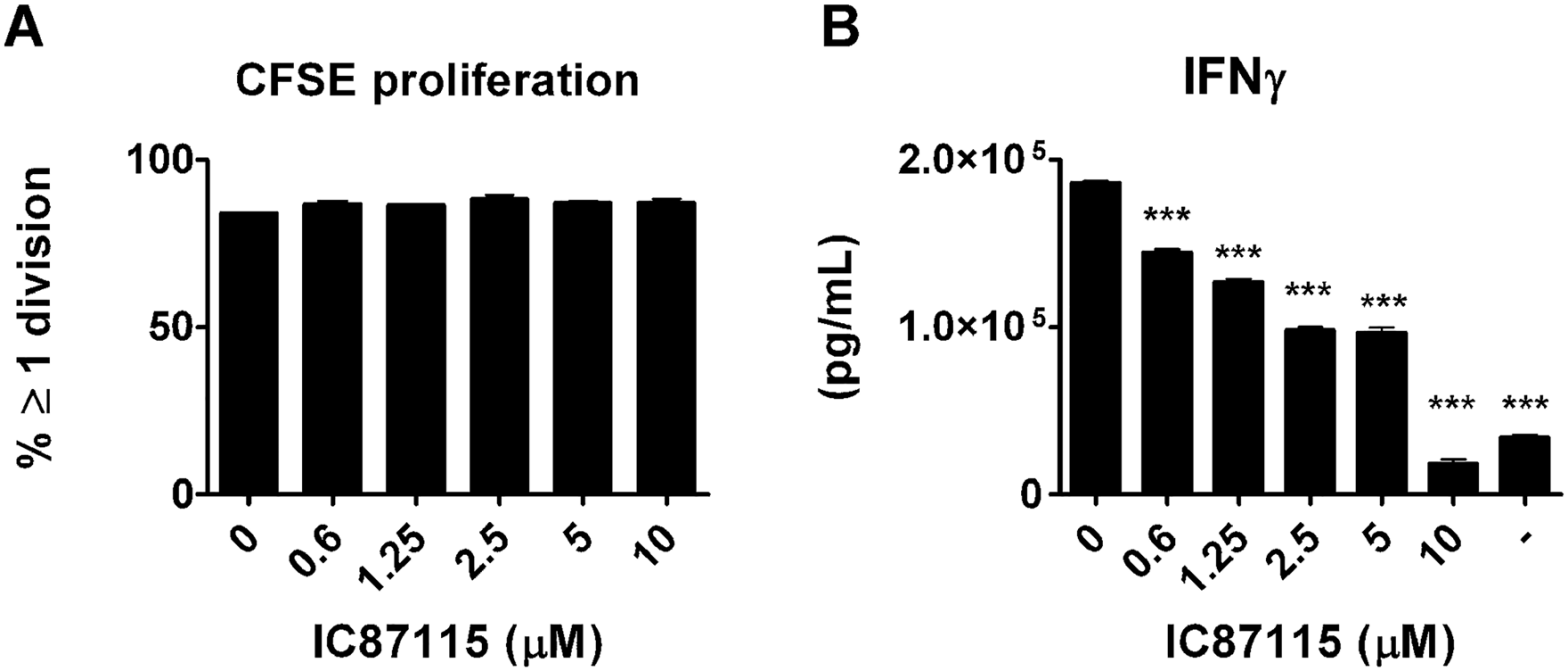
IC87114 reduces IFN-γ production by differentiated Th1 cells. Cells isolated from the spleens and lymph nodes of BDC2.5 TCR transgenic NOD mice were differentiated into Th1 cells by culturing them in the presence of anti-CD3/CD28 antibodies, IL-2, IL-12 and IFN-γ for 4 days. 2.5x10^5^ Th1 cells were then cultured with 1x10^4^ APCs and BDC2.5 mimotope (0.5 μg/mL) with or without increasing concentrations of IC87114 (0.6–10μM) for 72 hours. Cells were stained with CFSE and after gating on CD4^+^ T cells, the percent of proliferating cells was determined (A). Supernatants were collected and IFN-γ production was assessed by specific ELISA (B). All data were expressed as the mean ± SD for triplicate samples. The results are representative of at least three independent experiments, differences between groups were tested using the student t-test.

**Fig 4:**
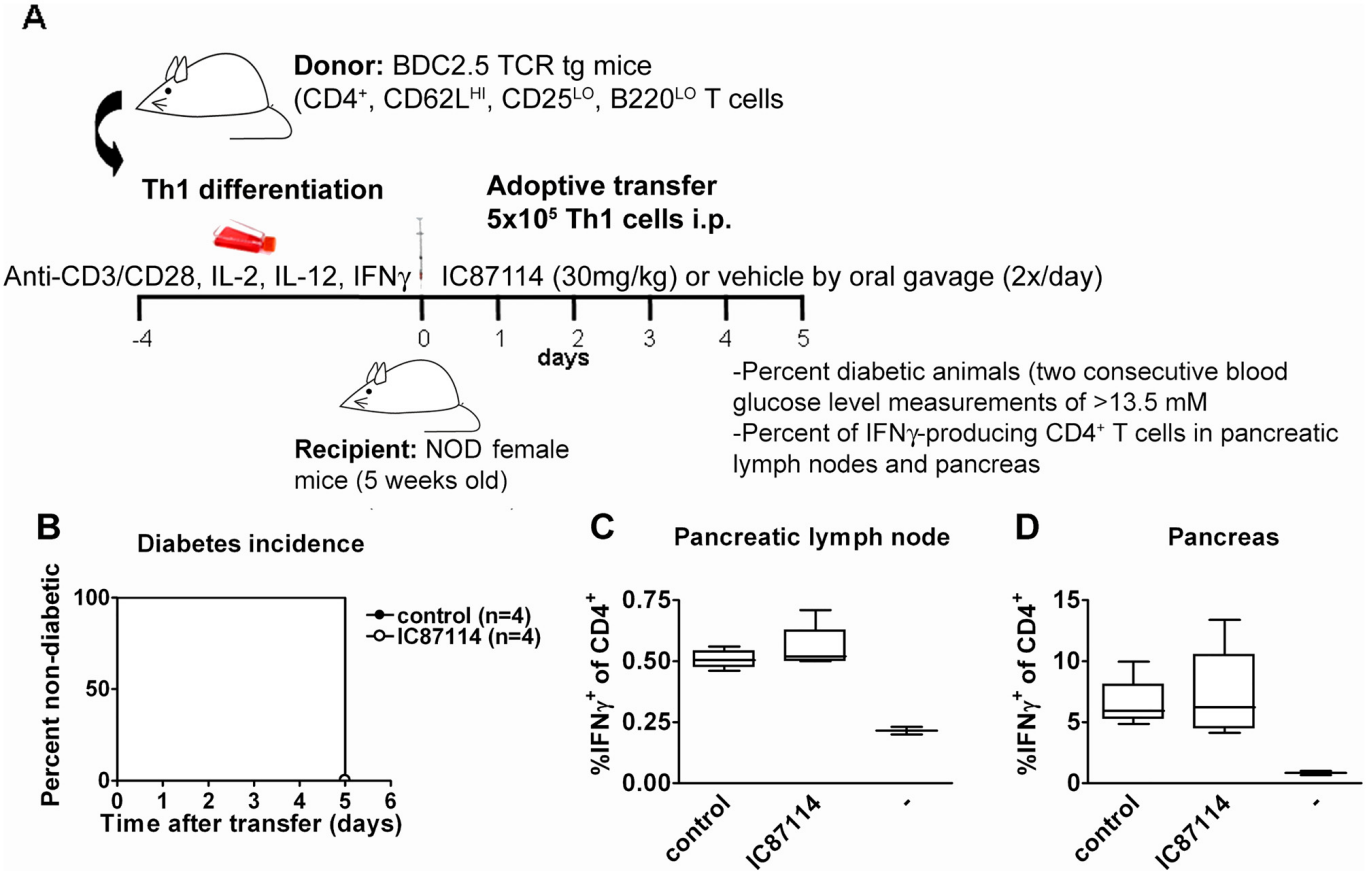
IC87114 treatment does not reduce the incidence of diabetes after adoptive transfer of *Th1* cells to wt NOD mice. CD4^+^, CD62L^hi^, CD25^lo^ B220 ^lo^ T cells were isolated by cell sorting from spleen and lymph nodes of BDC2.5 TCR transgenic mice and differentiated into Th1 cells by culturing them in the presence of anti-CD3/CD28 antibodies, IL-2, IL-12 and IFN-γ for 4 days. 5x10^5^ Th1 cells were transferred i.p. to wt NOD mice and IC87114 (30mg/Kg) or vehicle were administrated by oral gavage twice daily from day 0 to 5 (A). Mice were considered diabetic after 2 consecutive blood glucose readings > 13mM (B). We assessed the percentages of IFN-γ-producing CD4^+^T cells from pancreatic lymph nodes (C) and pancreas (D) in vehicle treated mice, IC87114 treated mice and negative control mice (4 mice per group). Negative control NOD mice did not receive Th1 cells nor any treatment.

As IC87114 did not have an effect on development of diabetes after transfer of BDC2.5 cells we wished to elucidate whether it could decrease IFN-γ production from already Th1 differentiated effector CD4^+^ T cells. We found that, just as seen in *in vitro* cultures with naïve cells, the Th1 differentiated effector cells proliferated equally well in the presence of IC87114 (Fig 3A), but that their IFN-γ production, although more robust than from naïve T cells, was decreased by IC87114 in a dose-dependent way (Fig 3B). Th1 differentiated effector BDC2.5 CD4^+^ T cells cause diabetes after transfer into wildtype NOD mice in a process heavily dependent on production of IFN-γ (Fig 4A) [30], and we hypothesised that administration of IC87114 via oral gavage which can stop IFN-γ production from these cells in vitro, would inhibit or delay onset of diabetes in the IFN-γ dependent model of disease. However, oral administration of 30mg/kg body weight twice per day from the day of cell transfer did not affect disease development, and both the IC87114-treated and the vehicle treated group developed diabetes on day 5 after adoptive transfer (Fig 4B). Oral administration did not appear to affect IFN-γ production *in vivo* as cells isolated from pancreas (Fig 4C) or pancreatic lymph nodes (Fig 4D) of IC87114 or vehicle-treated mice produced equal amounts of IFN-γ.

### Combination with CTLA4-Ig does not alter or potentiate the *in vitro* effects of IC87114

**Fig 5:**
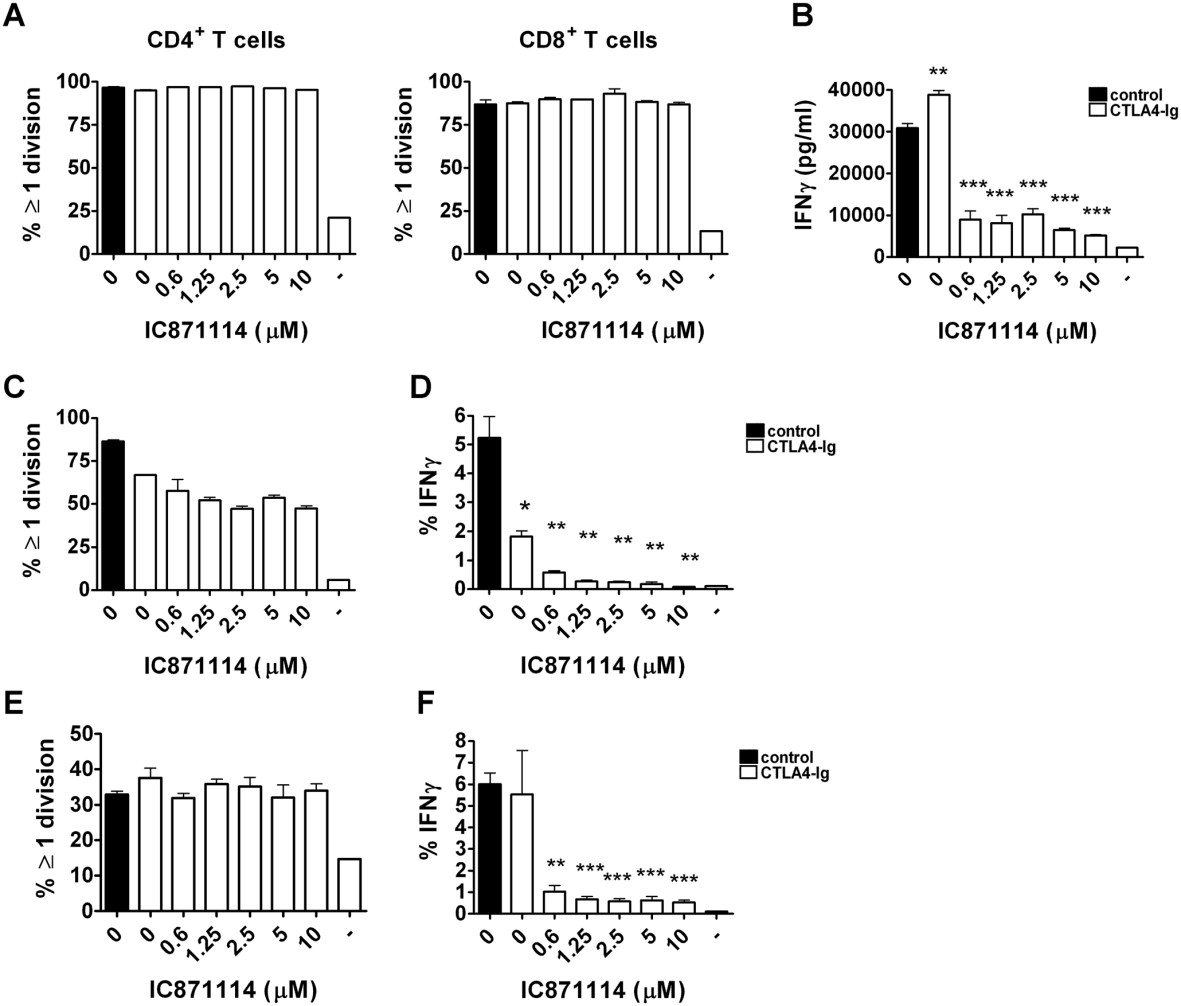
Combination of CTLA4-Ig with IC87114 has no major effect on diabetogenic cells. Cells isolated from the spleens and lymph nodes of wild-type NOD mice (A,B); BDC2.5 TCR transgenic NOD mice (C,D) or G9C8 TCR transgenic NOD mice (E,F) were stimulated with anti-CD3/28 antibodies (A, B), BDC2.5 mimotope (C, D) or insulin peptide (E, F), respectively, with or without increasing concentrations of IC87114 (0.6–10μM), with or without CLTA4-Ig (100 ng/ml) as indicated, for 72 hours. Cells were stained with CFSE and after gating on CD4^+^ and/or CD8^+^ T cells, the percent of proliferating cells in each population was determined (A,C,E). IFN-γ production was assessed either through ELISA (B) or intracellular staining (D, F). All data were expressed as the mean ± SD for triplicate samples, differences between groups were tested using the student t-test. The data is representative of at least three independent experiments.

As IC87114 showed no effect on *in vivo* diabetes development despite its dramatic effects on cytokine production *in vitro*, we hypothesised that a combination with another agent that targets a different pathway leading to the activation of T cells might synergise with the IC87114. Indeed, in work leading up to the current study, we found that p110δ-CD28 double knockout mice are immune suppressed and failed to reject allogeneic islet grafts ([20] and S4 Fig). Since CTLA-4 Ig inhibits the ability of T cells to receive signals via CD28, we assessed the effects of combining increasing levels of IC87114 with CTLA4-Ig on T cells from NOD mice in *in vitro* culture, looking at anti-CD3 induced proliferation of CD4^+^ T cells (Fig 1A, left hand panel) and CD8^+^ T cells (Fig 1A, right hand panel) and production of IFN-γ (Fig 1B). We found that the combination of CTLA4-Ig with IC87114 did not suppress proliferation of either CD4^+^ or CD8^+^ T cells any differently than IC87114 on its own (Fig 5A) and that the production of IFN-γ was similarly impaired in the presence of CTLA4-Ig (Fig 5B) compared with IC87114 alone (Fig 1B). The presence of CTLA4-Ig modestly impaired proliferation of BDC2.5 CD4^+^ T cells, but this was not potentiated by increasing concentrations of IC87114 (Fig 5C). However, CTLA4-Ig did have a significant effect on the production of IFN-γ from the BDC2.5 CD4^+^ T cells, and combination with IC87114 decreased IFN-γ production even further (Fig 5D). The proliferation and IFN-γ production of G9C8 CD8^+^ T cells was not affected by the addition of CTLA4-Ig (Fig 5D and 5E), demonstrating the same reduction of IFN-γ production in response to IC87114 as when no CTLA4-Ig was present (Fig 1F). We performed an extended analysis of the effects on cytokine production by combining CTLA4-Ig and IC87114, and found that addition of CTLA4-Ig on its own decreased the production of IL-2 in supernatants from peptide-stimulated BDC2.5 CD4^+^ T cells, but that other cytokines assessed demonstrated no additional downregulation in response to the additional presence of CLTA4-Ig to the increasing concentrations of IC87114 in the culture (S5 Fig). We did not detect an effect of CTLA-4 Ig with the bead array, which could be due to the fact that the high concentration of IFN-γ in the samples exceeding the top limit of the assay (S5 Fig).

### Combination treatment with CTLA4-Ig does not increase protection from diabetes by IC87114

**Fig 6:**
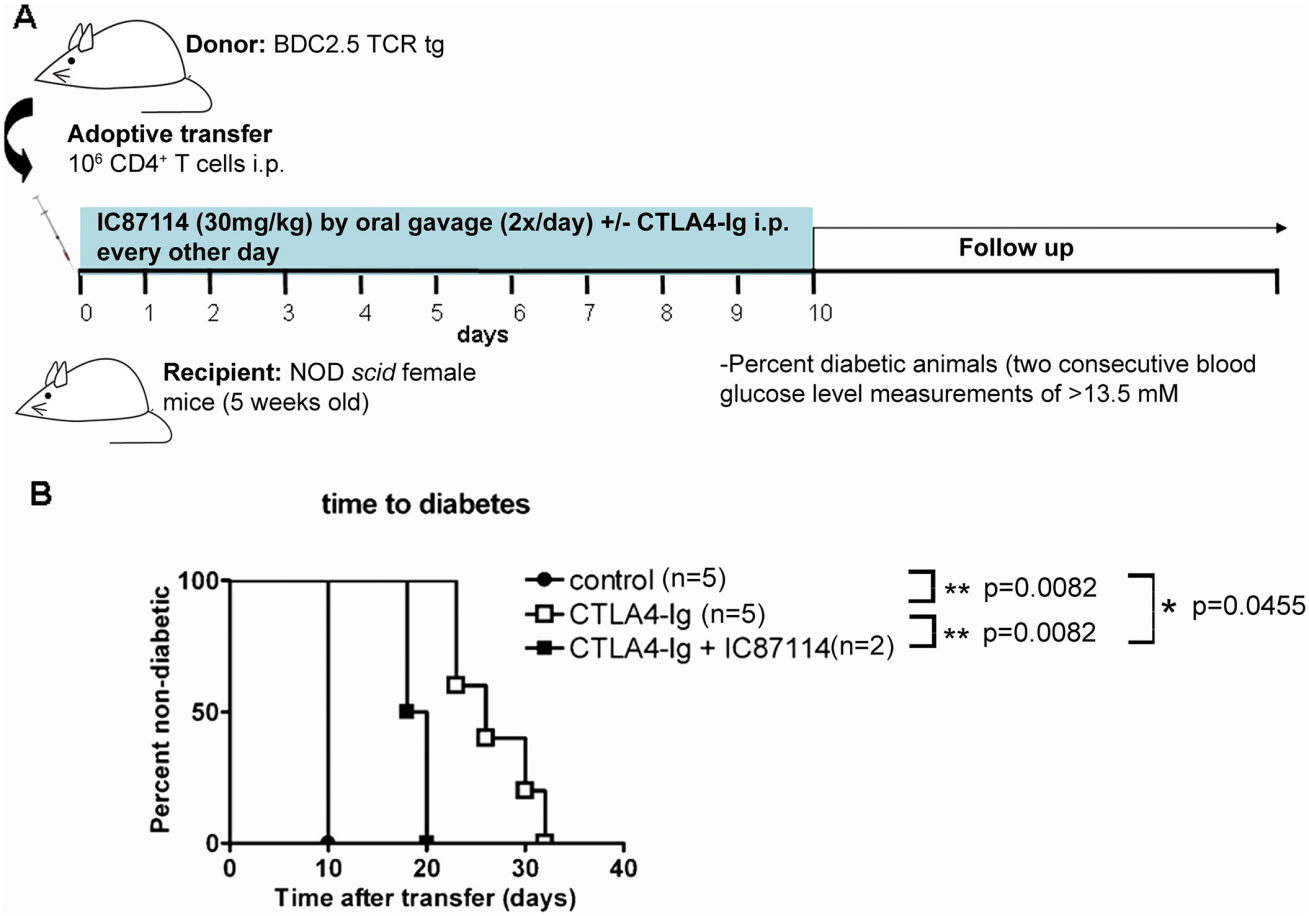
Combination treatment with CTLA4-Ig and IC87114 does not prevent diabetes after adoptive transfer of naïve diabetogenic cells. CD4^+^ T cells were isolated by cell sorting from spleen and lymph nodes of BDC2.5 TCR transgenic mice. 1x10^6^ CD4^+^ T cells were transferred i.p. route to NOD-*scid* mice. Mice received IC87114 treatment (30mg/Kg) or vehicle by oral gavage (2x/day) from day 0 to 10, and/or ip injections of CTLA4-Ig on day 0, 3 and 5 (A). Positive control mice received CD4^+^ T cells from BDC2.5 mice without any other treatment. Blood glucose levels were checked every day from day 7, and mice were considered irrevocably diabetic and sacrificed when they reached two consecutive blood glucose levels >13mM (B). Differences between treatment groups were determined using the Log Rank survival test.

As production of IFN-γ was further decreased in BDC2.5 CD4^+^ T cells *in vitro* when CTLA4-Ig was added to IC87114 in culture, we tested whether oral administration of IC87114 in combination with CTLA4-Ig could prevent or delay onset of diabetes in NOD *scid* mice after transfer of BDC2.5 CD4^+^ T cells (Fig 6A). We found that intraperitoneal injections of CTLA4-Ig every other day for the first 10 days after transfer significantly delayed onset of diabetes in recipient mice (Fig 6B), but that combination with twice daily administration of IC87114 did not enhance the protective effect, but rather appeared to attenuate it (Fig 6B).

### Combination with CTLA4-Ig does not alter or potentiate the *in vitro* or *in vivo* effects of IC87114 on Th1 differentiated effector T cells

**Fig 7:**
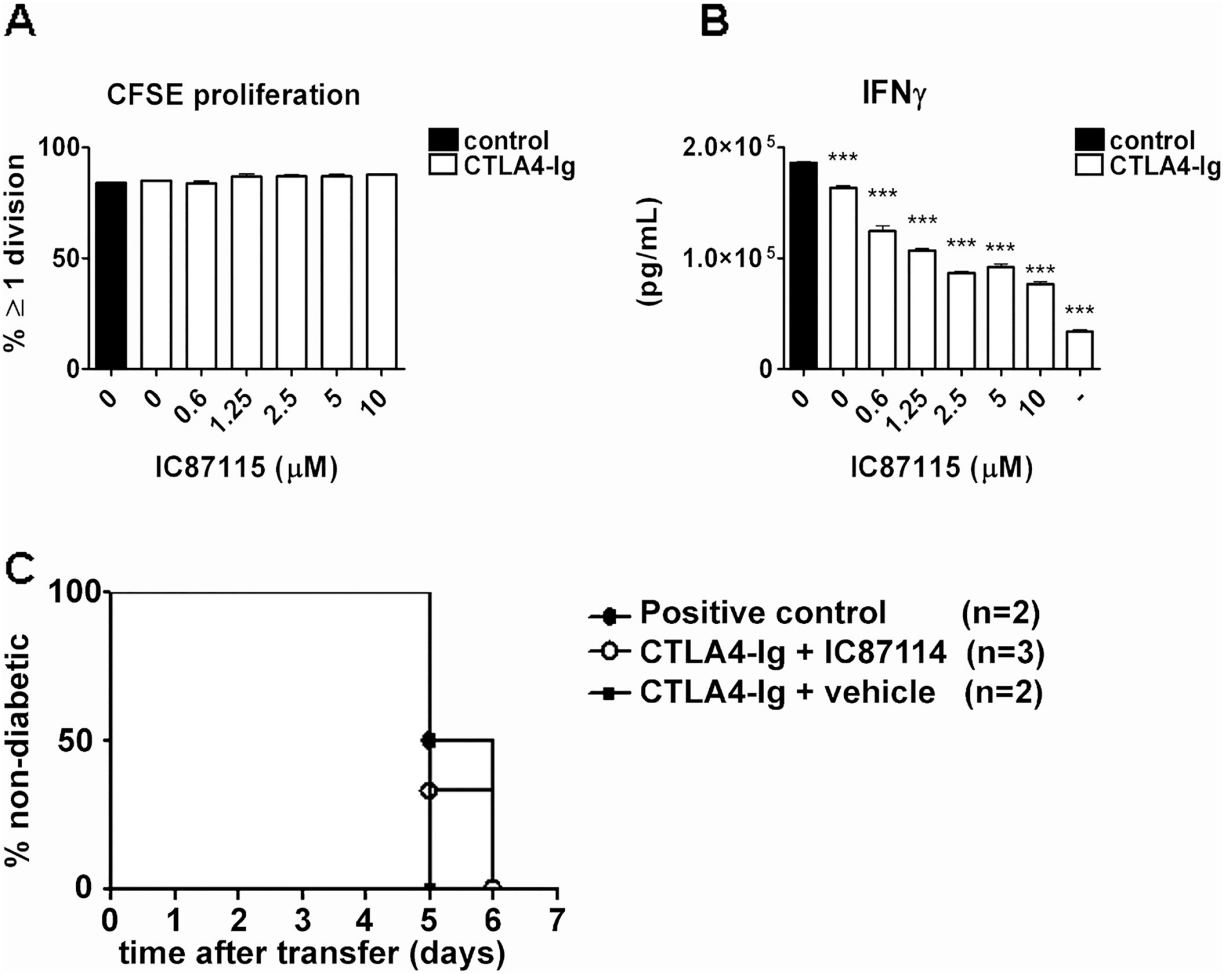
Combined treatment of IC87114 and CTLA-4 Ig does not reduce the incidence of diabetes in the adoptive transfer model of *Th1* cells from BDC2.5 transgenic mice to NOD mice. CD4^+^, CD62L^hi^, CD25^lo^ B220 ^lo^ T cells were isolated by cell sorting from spleen and lymph nodes of BDC2.5 TCR transgenic mice and differentiated into Th1 cells by culturing them in the presence of anti-CD3/CD28 antibodies, IL-2, IL-12 and IFN-γ for 4 days. Th1 cells were then cultured with 1x10^4^ APCs and BDC2.5 mimotope (0.5 μg/mL) with or without increasing concentrations of IC87114 (0.6–10μM)), with or without CTLA4-Ig (100 ng/ml) as indicated, for 72 hours. Cells were stained with CFSE and after gating on CD4^+^ T cells, the percentage of proliferating cells was determined (A). Supernatant IFN-γ assessed by specific ELISA (B). All data expressed as the mean ± SD for triplicate samples, differences between groups were tested using the student t-test. The results are representative of at least three independent experiments. 5x10^5^ Th1 cells were transferred i.p. to wt NOD mice. IC87114 (30mg/Kg) or vehicle were administrated by oral gavage twice daily from day 0 to 5, and/ CTLA4-Ig injected ip on day 0, 3 and 5. The number of mice per group is indicated within the Fig Mice were considered diabetic after 2 consecutive blood glucose readings > 13mM (C).

We also assessed whether addition of CTLA4-Ig had an additional inhibitory affect on IFN-γ production from already Th1 differentiated effector CD4^+^ T cells. We found that, just as seen in Fig 3, that the Th1 differentiated effector cells proliferated equally well in the presence of IC87114 (Fig 3A), but that their IFN-γ production, although more robust than from naïve T cells, was decreased by IC87114 in a dose dependent way. Addition of CTLA4-Ig to the culture had no effect on proliferation (Fig 7A), and had some effect on IFN-γ production, not appearing to result in synergistic suppression (Fig 7B). Neither treatment with CTLA4-Ig on its own nor in combination with IC87114 affected development of diabetes after transfer of Th1 differentiated effector BDC2.5 CD4^+^ T cells into wt NOD mice (Fig 7C), as all mice regardless of treatment developed diabetes 5 to 6 days after injection of the diabetogenic cells.

### IC87114 does not alter the percentage of regulatory T cells, but reduces their production of IL-10

**Fig 8:**
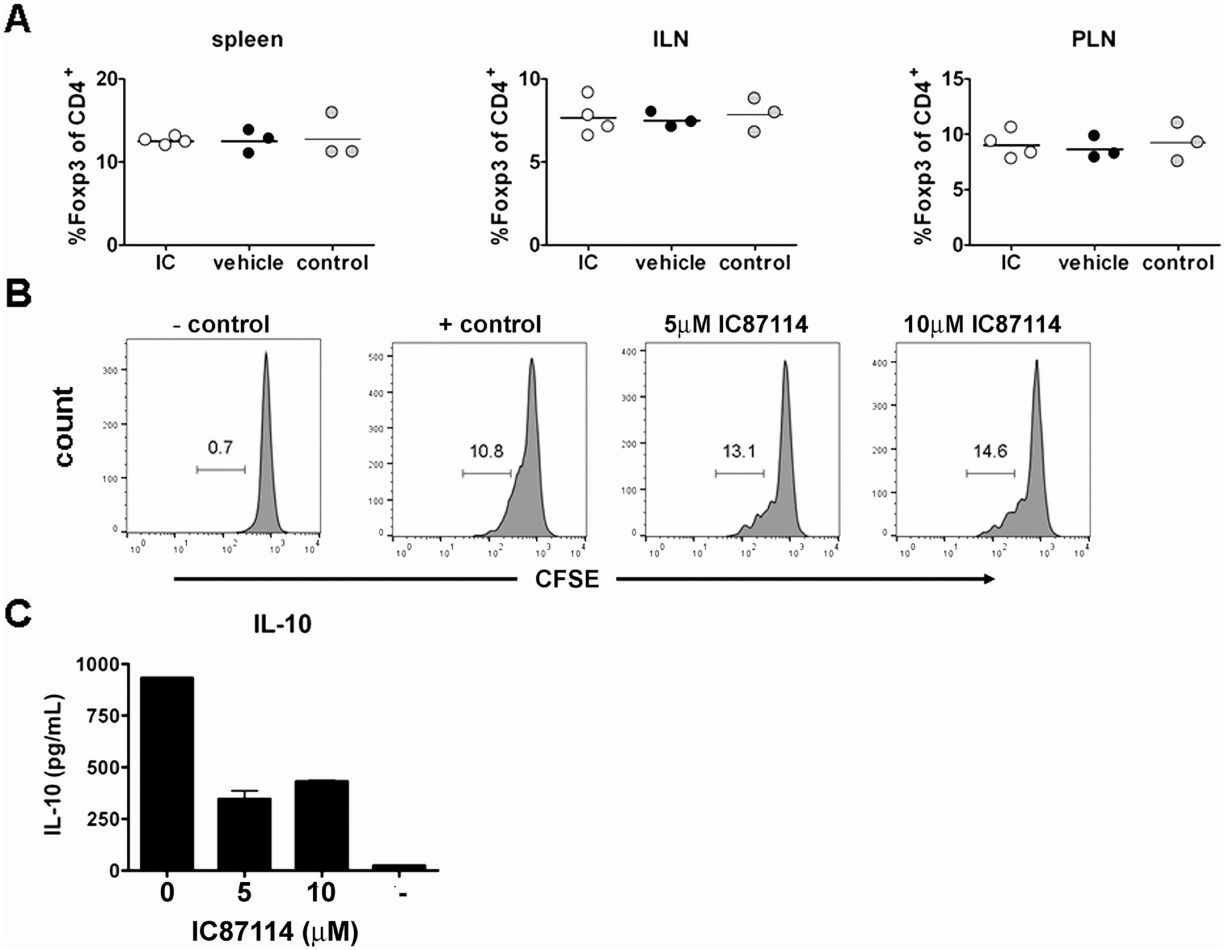
IC87114 does not affect percentages of Foxp3^+^ Treg in treated mice, but reduces IL-10 production from Treg. Percentages of Foxp3^+^ cells were assessed in spleen, inguinal lymph nodes and pancreatic lymph nodes of mice treated with IC87114 twice daily for 10 days. Each data point represents one mouse (A). Proliferation of sorted CFSE labeled BDC2.5 Foxp3-GFP cells in response to anti-CD3/CD28 antibodies in the presence of IC87114 (B). Production of IL-10 from stimulated, sorted, BDC2.5 Foxp3-GFP in presence of IC87114 measured by supernatant ELISA (C). Results are representative of at least two experiments.

Treatment with IC87114 appeared to break the temporary tolerance afforded by CTLA4-Ig treatment after islet specific T cell transfer (Fig 6B). As the effects of IC87114 on the cytokine production of these cells were so striking, we wondered if effects of IC87114 on the regulatory T cell pool could be even more important, and thus cancel out and even override any anti-inflammatory effects. We found that oral administration of IC87114 had no effect on the percentages of Treg present in the spleen, inguinal or pancreatic lymph nodes of treated mice (Fig 8A) or on their absolute numbers (data not shown), and that presence of IC87114 in culture medium did not affect proliferation of sorted Treg from Foxp3-GFP reporter NOD mice *in vitro* (Fig 8B). However, we found that cytokine production was strongly affected by IC87114, demonstrating a ~65% drop in IL-10 production in the presence of 5mM IC87114 (Fig 8C).

## Discussion

IC87114 has a profound suppressive effect on the production of proinflammatory cytokines such as IFN-γ and IL-17 as well as IL-2 from islet-specific cells activated *in vitro*, while increasing the production of anti-inflammatory cytokines such as IL-4 and IL-10. As the development of diabetes in NOD mice depends on the production of IFN-γ from CD4^+^ T cells [30], and IFN-γ production and IL-17 production is elevated in islet-reactive CD4^+^ T cells in recent onset type 1 diabetes patients [5, 31], we hypothesised that administration of IC87114 could have beneficial effects on anti-islet inflammation and be a feasible option of type 1 diabetes therapy. A previous study has demonstrated modest positive effects of IC87114 on the development of diabetes in NOD mice [32], and we hypothesised that combination of this agent which has such dramatic effects on production of pro-inflammatory cytokines even from already activated cells [15] with another well tolerated treatment targeting the activation of T cells [11, 26] would have a chance of preventing disease completely. Indeed, preliminary studies showed that genetic inactivation of PI3Kδ and CD28 resulted in indefinite acceptance of islet allografts.

However, we found that, despite seeing considerable inhibition of pro-inflammatory cytokine production even at the lowest concentrations of IC87114 tested, treatment with IC87114 had no effect on the development of diabetes either after adoptive transfer of naïve BDC2.5 CD4^+^ T cells into NOD *scid* mice or after transfer of Th1 differentiated effector BDC2.5 CD4^+^ T cells into wild type NOD mice. The group sizes we used for *in vivo* experiments were small, and it cannot be excluded that differences between groups could have been detected had the sample size been larger. However, as no positive effects of IC87114 were detected in the preliminary *in vivo* experiments, we decided against using larger numbers of mice which might have allowed us to detect subtle differences between groups.

Adding CTLA4-Ig to cultures had specific effects, targeting CD4^+^ T cell proliferation and production of IL-2 from these cells, and combination of CLTA4-Ig with IC87114 had a major effect on IFN-γ production from CD4^+^ T cells. This was considered promising as IFN-γ production from CD4^+^ T cells that has been shown to be particularly important in the pathology of diabetes in mice as well as men [5, 30]. However, combination treatment with twice daily oral administration of IC87114 and intraperitoneal injections with CTLA4-Ig every other day for 10 days did not lead to protection from diabetes either after transfer of naïve cells or pre-activated Th1 cells. In fact, treatment with IC87114 appeared to attenuate the protective effect afforded by CTLA4-Ig injection, raising the question of whether effects on the regulatory T cell subset may be greater than any effects on anti-inflammatory cytokine production. We found that although IC87114 treatment did not change the percentage of Foxp3^+^ regulatory CD4^+^ T cells present in secondary lymphoid organs, these cells produced less anti-inflammatory IL-10 when activated in the presence of IC87114, and are thus also a feasible targets of IC87114 treatment. Production of anti-inflammatory cytokines such as IL-10 [33] and TGF-β is known to suppress development of diabetes in NOD mice [34, 35], and it is not surprising that a reduction in the production of anti-inflammatory cytokines could precipitate disease.

Regulatory T cells have been identified as a target of PI3K p110δ inhibitors, and targeting p110δ signalling in these cells increases anti-tumour immune responses by releasing cytotoxic CD8^+^ T cells from Treg control [36, 37] even though lack of p110δ signalling also resulted in decreased CD8^+^ T cell expression of activation marker CD44 as well as granzyme B and perforin. Our findings demonstrate major effects of IC87114 on the production of pro-inflammatory cytokines from diabetogenic T cell clones *in vitro*, but no decrease in their diabetogenicity *in vivo*. In fact, we see that treatment with IC87114 abrogates the protective effects of CTLA4-Ig treatment and causes a quicker progression to diabetes after cell transfer. Our results indicate that IC87114 is not a promising candidate for treatment of type 1 diabetes, even in combination with other drugs such as CTLA4-Ig, but that it leaves the *in vivo* inflammatory response unchanged. Instead, our data adds weight to the proposal that PI3K p110δ inhibition, as a means to reduce the effect of Treg, is a feasible strategy for breaking tumour-specific immune tolerance to achieve improved cancer immunotherapy.

## Supporting Information

S1 Fig Effects of IC87114 on cytokine production in BDC2.5 CD4^+^ T cells. Cells isolated from the spleens and lymph nodes of BDC2.5 TCR transgenic NOD mice were stimulated with the BDC2.5 mimotope (0.5 μg/mL) with or without increasing concentrations of IC87114 (0.6–10μM) for 48 hours. Cytokines from supernatants were assessed in duplicate using a bead cytokine array, differences between groups were tested using the student t-test.
(TIF)

S2 Fig Effects of IC87114 on cytokine production in G9C8 CD8^+^ T cells. Cells isolated from the spleens and lymph nodes of G9C8 TCR transgenic NOD mice were stimulated with the insulinB 15–23 peptide (0.5 μg/mL) with or without increasing concentrations of IC87114 (0.6–10μM) for 48 hours. Cytokines from supernatants were assessed in duplicate using a bead cytokine array, differences between groups were tested using the student t-test.
(TIF)

S3 Fig Effects of IC87114 on the distribution of divisions and activation status. Cells isolated from the spleens and lymph nodes of BDC2.5 TCR transgenic NOD mice were stimulated with the BDC2.5 mimotope (0.5 μg/mL) with or without increasing concentrations of IC87114 (0.6–10μM) for 72 hours, and then stained for CD25 A histogram overlay of representative cultures gated on CD4^+^ cells (A, left) and a graph showing all data (A, right). Cells isolated from the spleens and lymph nodes of G9C8 TCR transgenic NOD mice were stained with CFSE and stimulated with the insulinB 15–23 peptide (0.5 μg/mL) with or without increasing concentrations of IC87114 (0.6–10μM) for 72 hours. A histogram overlay of representative cultures gated on CD8^+^ cells (B, left), and a graph showing all data (B, right). Differences between groups were tested using the student t-test.
(TIF)

S4 Fig Survival of MHC mis-matched islets in streptozotocin induced diabetic recipients. Wt C57BL/6 mice, CD28 KO, PI3K p110D910A (D910A) and CD28-D910A double deficient mice (DKO) were rendered diabetic through injection of streptozotocin. Diabetic mice received a MHC mis-matched (Cba1-C57BL/6 F1 donor) islet graft under the kidney capsule. Blood glucose was monitored in the recipient mice for up to 215 days. Some DKO mice that remained euglycemic for a long time underwent nephrectomy at the end of the experiment to ascertain that the graft was the cause of the restored euglycemia. The difference in euglycemic survival between wt recipient mice and DKO recipient mice was assessed using the Log Rank survival test, resulting in a p-value of 0.0027 (**).
(TIF)

S5 Fig Effects of combination of CTLA4-Ig and IC87114 on cytokine production in BDC2.5 CD4^+^ T cells. Cells isolated from the spleens and lymph nodes of BDC2.5 TCR transgenic NOD mice were stimulated with the BDC2.5 mimotope (0.5 μg/mL) in the presence of CTLA4-Ig (100 ng/mL) with or without increasing concentrations of IC87114 (0.6–10μM) for 48 hours. Cytokines from supernatants were assessed in duplicate using a bead cytokine array, differences between groups were tested using the student t-test.
(TIF)
