# Supplementary figures and images for: Inhibition of Phosphoinositide 3-Kinase p110delta Does Not Affect T Cell Driven Development of Type 1 Diabetes Despite Significant Effects on Cytokine Production
Source: PLoS One. 2016 Jan 19;11(1):e0146516. doi: 10.1371/journal.pone.0146516 (PMC4718552; doi:10.1371/journal.pone.0146516)

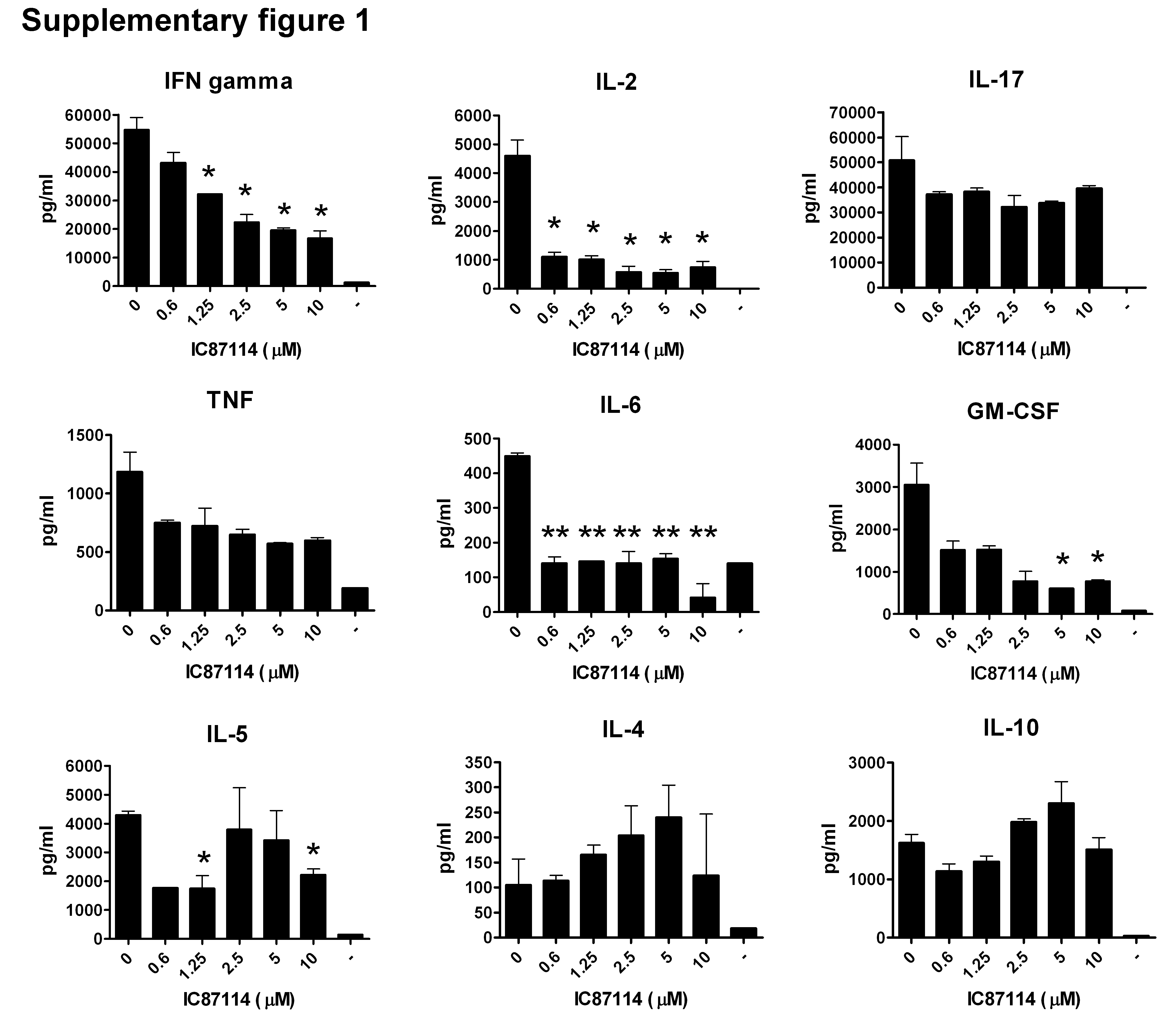

Supplement: S1 Fig — Cells isolated from the spleens and lymph nodes of BDC2.5 TCR transgenic NOD mice were stimulated with the BDC2.5 mimotope (0.5 μg/mL) with or without increasing concentrations of IC87114 (0.6–10μM) for 48 hours. Cytokines from supernatants were assessed in duplicate using a bead cytokine array, differences between groups were tested using the student t-test. (TIF) [file pone.0146516.s001.tif]

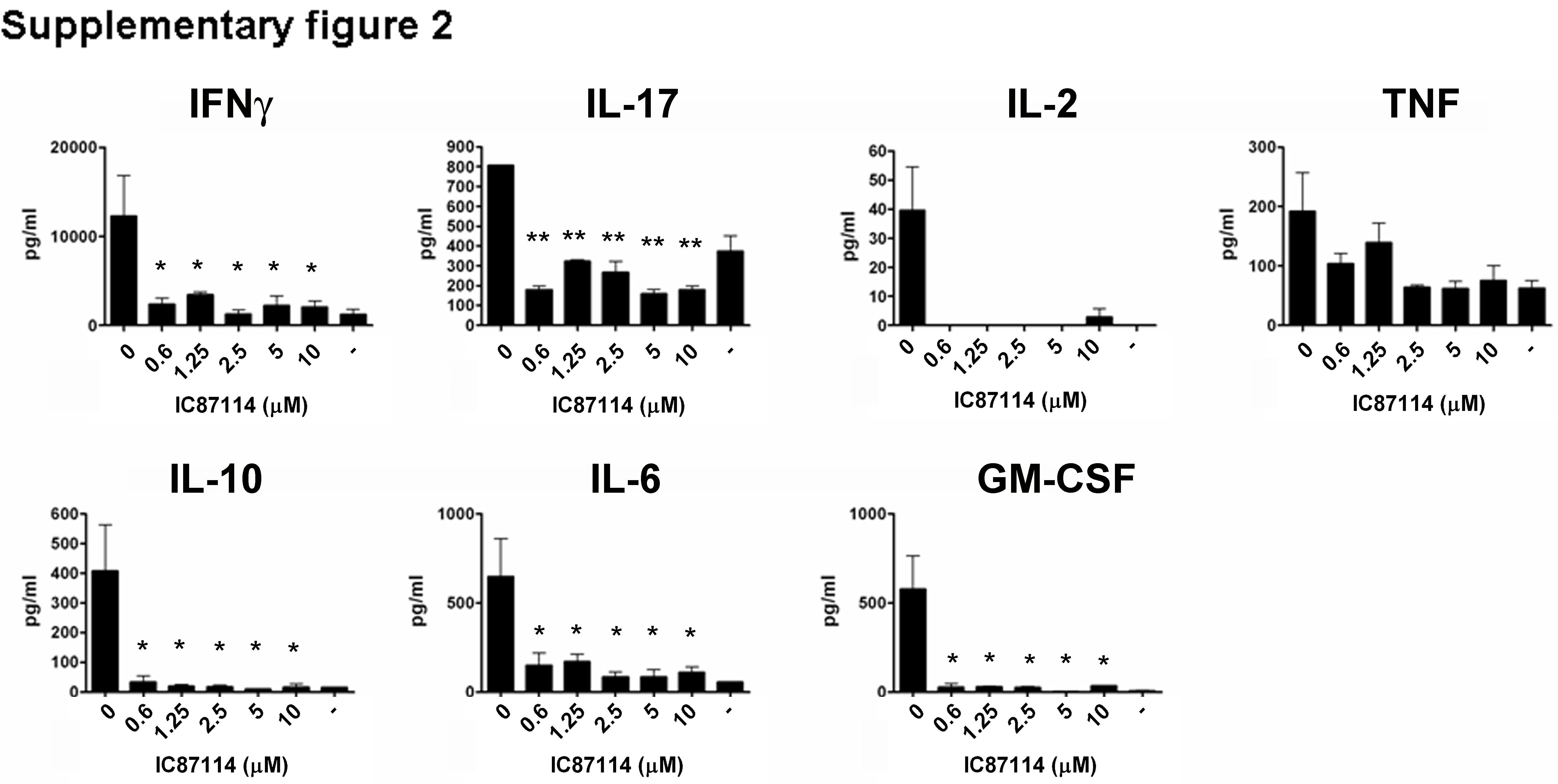

Supplement: S2 Fig — Cells isolated from the spleens and lymph nodes of G9C8 TCR transgenic NOD mice were stimulated with the insulinB 15–23 peptide (0.5 μg/mL) with or without increasing concentrations of IC87114 (0.6–10μM) for 48 hours. Cytokines from supernatants were assessed in duplicate using a bead cytokine array, differences between groups were tested using the student t-test. (TIF) [file pone.0146516.s002.tif]

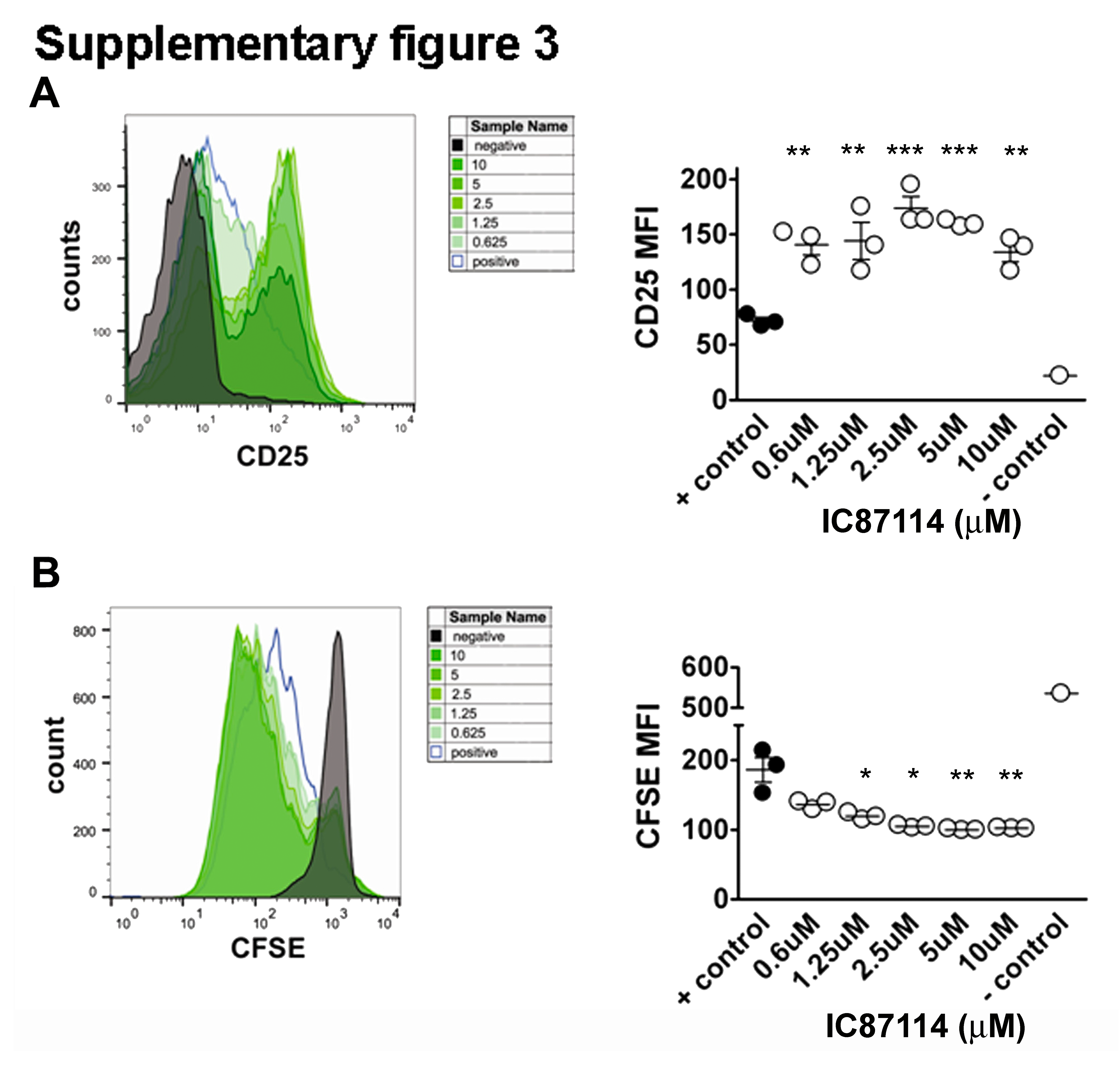

Supplement: S3 Fig — Cells isolated from the spleens and lymph nodes of BDC2.5 TCR transgenic NOD mice were stimulated with the BDC2.5 mimotope (0.5 μg/mL) with or without increasing concentrations of IC87114 (0.6–10μM) for 72 hours, and then stained for CD25 A histogram overlay of representative cultures gated on CD4+ cells (A, left) and a graph showing all data (A, right). Cells isolated from the spleens and lymph nodes of G9C8 TCR transgenic NOD mice were stained with CFSE and stimulated with the insulinB 15–23 peptide (0.5 μg/mL) with or without increasing concentrations of IC87114 (0.6–10μM) for 72 hours. A histogram overlay of representative cultures gated on CD8+ cells (B, left), and a graph showing all data (B, right). Differences between groups were tested using the student t-test. (TIF) [file pone.0146516.s003.tif]

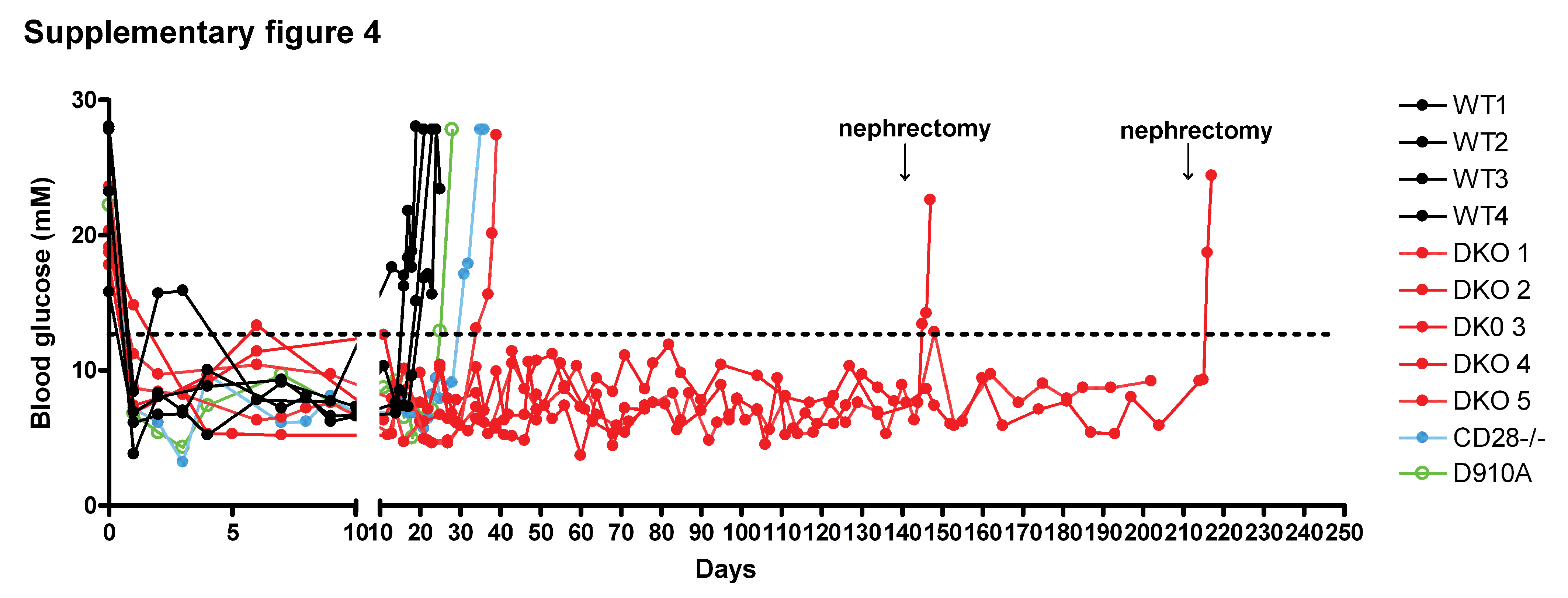

Supplement: S4 Fig — Wt C57BL/6 mice, CD28 KO, PI3K p110D910A (D910A) and CD28-D910A double deficient mice (DKO) were rendered diabetic through injection of streptozotocin. Diabetic mice received a MHC mis-matched (Cba1-C57BL/6 F1 donor) islet graft under the kidney capsule. Blood glucose was monitored in the recipient mice for up to 215 days. Some DKO mice that remained euglycemic for a long time underwent nephrectomy at the end of the experiment to ascertain that the graft was the cause of the restored euglycemia. The difference in euglycemic survival between wt recipient mice and DKO recipient mice was assessed using the Log Rank survival test, resulting in a p-value of 0.0027 (**). (TIF) [file pone.0146516.s004.tif]

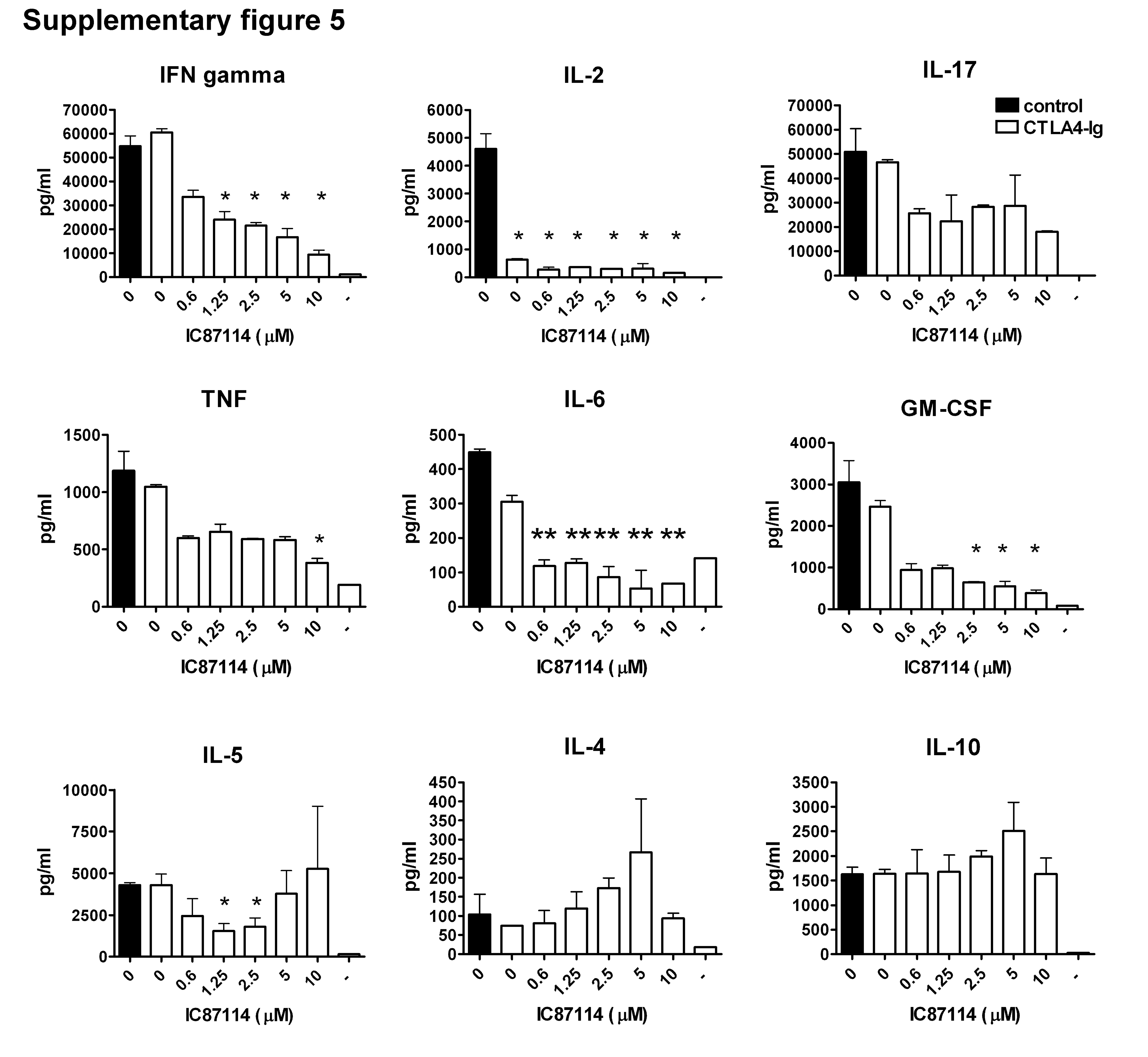

Supplement: S5 Fig — Cells isolated from the spleens and lymph nodes of BDC2.5 TCR transgenic NOD mice were stimulated with the BDC2.5 mimotope (0.5 μg/mL) in the presence of CTLA4-Ig (100 ng/mL) with or without increasing concentrations of IC87114 (0.6–10μM) for 48 hours. Cytokines from supernatants were assessed in duplicate using a bead cytokine array, differences between groups were tested using the student t-test. (TIF) [file pone.0146516.s005.tif]
